# Seasonal and Species-Specific Variations in Gut Microbiota of Wild Ungulates in Captivity

**DOI:** 10.3390/ani16101437

**Published:** 2026-05-08

**Authors:** Hongrui Lin, Shuang Cui, Shuxiang Hao, Wei Yu, Miao Jin, Tingwen Jin, Xia Ren, Jinlian Ma, Suhe Gu, Liwei Teng, Zhensheng Liu

**Affiliations:** 1 College of Wildlife and Protected Areas, Northeast Forestry University, Harbin 150040, China; hongruilin@nefu.edu.cn (H.L.); cs1374789090@163.com (S.C.); weiyu0603@126.com (W.Y.); pearsonj@163.com (M.J.); 2 Helan Mountain National Nature Reserve of Inner Mongolia, Alxa East County 750306, China; zqynhsx@163.com (S.H.); azqlyjjtw@163.com (T.J.); 13948023111@163.com (X.R.); 13384839891@163.com (J.M.); haojuan145@126.com (S.G.)

**Keywords:** captive ungulates, gut microbiota, seasonal variations, species-specific differences, captive conditions

## Abstract

Seasonal variation can strongly restructure the gut microbiota of herbivores, but under captive conditions it remains unclear whether these shifts outweigh host-species effects when diet is standardized. Here, we profiled the gut microbiota of captive mouflon, argali, and blue sheep in summer and winter using 16S rRNA amplicon sequencing, ASV-based compositional beta-diversity analysis, ANCOM-BC2, and PICRUSt2 functional prediction. Across hosts, Chao1 richness decreased in winter, whereas Shannon diversity remained comparatively stable, indicating that seasonal change affected richness more strongly than evenness. CLR-based ordination further showed that the three species were relatively close in summer but became more clearly separated in winter, especially mouflon and blue sheep, consistent with pronounced seasonal restructuring under a common diet. At the phylum level, summer communities were broadly similar and dominated by Firmicutes_A and Bacteroidota, whereas winter brought stronger taxonomic shifts in mouflon and blue sheep, including marked increases in Proteobacteria and Firmicutes_D. Argali showed a narrower and taxonomically different seasonal response. Genus-level analyses similarly indicated that winter restructuring was host-dependent rather than uniform, with winter-associated enrichment of several Bacillales/Planococcaceae-related lineages and Proteobacteria-associated taxa, particularly in mouflon and blue sheep. Predicted functional profiles inferred by PICRUSt2 also shifted between summer and winter within each host species, suggesting that seasonal restructuring extended beyond taxonomy, although these results should be interpreted as inferred functional potential rather than direct metabolic activity. Overall, our findings support seasonality as the primary axis of gut microbiota variation in captivity, while the host species modulates the magnitude and taxonomic features of these seasonal changes. These results highlight the need to consider seasonal effects in captive management and provide a basis for future multi-omics validation of host-microbiome interactions in wild ungulates.

## Simple Summary

Gut microbiota in captive animals can still change markedly across seasons, even when animals are maintained under the same feeding regime. In this study, we compared the gut microbiota of three captive wild sheep species mouflon, argali, and blue sheep in summer and winter. We found that microbial richness declined in winter, while overall community structure was strongly reorganized. The three species showed relatively similar gut microbial communities in summer, but they became more differentiated in winter, especially in mouflon and blue sheep. Seasonal changes were therefore stronger than species differences overall, although host species still influenced the magnitude and taxonomic pattern of winter restructuring. These results indicate that seasonal conditions should be considered in captive animal management, even when diet is standardized, and that microbiome-informed management may help improve animal welfare and conservation practice.

## 1. Introduction

The gut microbiota is a diverse colony of bacteria that exists in the intestines of animals. It primarily relies on the host’s intestinal environment, constituting a critically important microbial community within the organism. This community is vital for several physiological and metabolic processes, including immunological defense, digestion, and nutrient synthesis [1,2,3]. It can also regulate host intestinal motility and secretion, protect the intestinal epithelial barrier, and participate in the digestion, absorption, and breakdown of macromolecules like polysaccharides in food [4]. In addition, dysbiosis of the gut microbiota poses a significant challenge in the management of captive and endangered species, as it can lead to health complications such as gastrointestinal disorders, reduced immune function, and impaired nutrient absorption [5]. It can not only induce various diseases, including gastrointestinal disorders, obesity, and their associated complications [6,7], but it may also impact the host’s behavior and ecological niche [8,9]. As a result, investigating the gut microbiota is an important route to improving our understanding of animal ecology, evolution, and conservation, especially for the preservation of endangered species [5].

Several factors determine the makeup of the animal gut microbiota, including host genetics, nutrition, and environmental variables such as temperature and season [10,11,12]. These factors are particularly important for herbivorous species, whose microbiota may vary with seasonal changes in food availability [13,14,15]. In particular, herbivorous animals’ gut symbiotic communities change in response to seasonal diet variations, increasing the host’s energy intake efficiency and maintaining greater seasonal physiological stability [16,17]. Seasonal restructuring of microbial communities in response to dietary fluctuations has been documented in species such as *Pseudois nayaur* [15] and *Bison bison* [13]. Moreover, seasonal climate changes also influence the composition of the gut microbiota [18]. All of this evidence indicates that seasonal factors might lead to the reconfiguration of the gut microbiota.

Understanding the co-evolution of gut microbial communities among various species is aided by comparing their gut microbiota. However, with the rapid expansion of manmade activity, a growing number of species face extinction, and captive breeding is increasingly being employed for conservation and reintroduction purposes [14]. Comparing the gut microbiota composition of various animals in captivity is therefore crucial. Simultaneously, functional data of the microbial community are critical for understanding the influence of microorganisms on host fitness. While the gut microbiota and its functional predictions have been researched in a few captive species, including the golden snub-nosed monkey and blue sheep [19], two important questions remain unresolved. First, it is still unclear whether season remains a major driver of gut microbiota variation when closely related herbivorous hosts are maintained under the same captive feeding regime. Second, it is unknown to what extent host species still modulates gut microbiota composition and predicted function under such standardized conditions. A comprehensive comparison of microbiota composition and function across multiple wild ungulate species under standardized captive conditions therefore remains lacking. To date, no study has simultaneously compared the gut microbiota composition and predicted functions among mouflon, argali, and blue sheep under controlled captive conditions. This knowledge gap limits our understanding of whether gut microbiota variation in captivity is driven primarily by seasonal change, by host species identity, or by their interaction.

Mouflon, argali, and blue sheep are three herbivorous ungulates belonging to the subfamily Caprinae of the family Bovidae [20]. Due to climate change, environmental alterations, intensified land use, and habitat degradation, the populations of these animals have sharply declined [21]. According to the IUCN Red List, mouflon and argali are classified as Near Threatened, whereas the blue sheep is listed as Least Concern [22]. In their wild habitats, these three species consume different plant species: during winter and summer, blue sheep predominantly feed on *Stipa* spp., *Poa* spp., and *Ulmus glaucescens*; argali primarily consume *Leymus chinensi* and *Cleistogenes squarrosa*; and grasses, forbs, and shrubs constitute the main food sources for mouflon. However, under captive conditions, these three species are provided the same diet in winter and summer, being fed a mixture of corn and local alfalfa hay. This shared feeding regime provides an opportunity to reduce dietary variation and more clearly examine the relative contributions of season and host species to gut microbiota variation in captivity.

We hypothesize that even under controlled captive conditions with a common diet, the gut microbiota composition will vary significantly between these species, influenced by both seasonal factors and host-specific factors. More specifically, we predict that (i) seasonal variation will remain a major driver of gut microbiota restructuring under captivity, and (ii) host species will modulate the magnitude and taxonomic characteristics of these seasonal shifts. This study compares the seasonal variations in the gut microbiota composition of mouflon, argali, and blue sheep under identical captivity conditions, aiming to identify species-specific microbial features and the impact of seasonal changes; explore the diversity and compositional differences in the gut microbiota among these species; and, based on these comparisons, analyze the main functions of these microbial communities. This research provides a theoretical basis for optimizing feeding strategies in captive management and enhances our understanding of the microbial ecology and microbial response patterns exhibited by different species in captivity. This study provides insights into the microbial ecological differences among different hosts, offering insights into the microbial ecology of captive mouflon, argali, and blue sheep. This information can also aid in implementing more effective conservation measures to preserve wildlife diversity.

## 2. Materials and Methods

### 2.1. Sample Collection

In the winter of 2020 (November–December) and the summer of 2021 (July–August), fresh fecal samples were collected from mouflon (summer: *n* = 7; winter: *n* = 6), argali (summer: *n* = 6; winter: *n* = 6), and blue sheep (summer: *n* = 6; winter: *n* = 10), which were fed the same diet, and kept at Zhongshan Park, Yinchuan City, China (Table S1). The animals were fed a controlled diet consisting of approximately 60% corn and 40% locally sourced alfalfa hay. The feeding regime remained consistent across seasons during the study period. Each fecal sample represented a single individual animal, and no individual was sampled more than once within the same season. Moreover, the individuals sampled in summer and winter were not the same animals. Therefore, all samples were treated as independent biological replicates in subsequent statistical analyses. All the blue sheep were rescued from the Helan Mountains and maintained in a controlled captive environment due to health conditions that prevented their release back into the wild. Fecal samples were promptly frozen at −80 °C after being properly collected to avoid contamination. Detailed information on individual animals, including sample ID, body weight, sex, and diet, is provided in Table S1.

### 2.2. Gut Microbiota Analysis

Total genomic DNA was extracted from fecal samples using the E.Z.N.A.™ Mag-Bind Soil DNA Kit (Omega Bio-Tek, Norcross, GA, USA) according to the manufacturer’s instructions. DNA concentration was measured using a Qubit 3.0 fluorometer (Invitrogen, Carlsbad, CA, USA). The V3-V4 region of the bacterial 16S rRNA gene was amplified using primers 338F (5′-ACTCCTACGGGGAGGAGCA-3′) and 806R (5′-GGACTACHVGGGTWTCTAAT-3′). PCR amplification and library construction were performed using a two-step PCR protocol with 2× Hieff^®^ Robust PCR Master Mix (Yeasen Biotechnology, Shanghai, China) in a total reaction volume of 30 µL. The first-round PCR was used to amplify the target region, and the second-round PCR was used to introduce Illumina-compatible index primers for library construction. PCR products were checked by agarose gel electrophoresis, and library concentration was quantified using a Qubit 3.0 fluorometer prior (Thermo Fisher Scientific, Carlsbad, CA, USA) to sequencing on the Illumina MiSeq platform (Illumina, Inc., San Diego, CA, USA). Raw reads were further subjected to bioinformatic quality control, including denoising and chimera removal, before downstream analyses. Because the wet-lab amplification and library preparation were performed by a commercial sequencing provider, and the available service documentation did not explicitly report negative or positive controls, their inclusion could not be verified retrospectively. Raw reads were imported into QIIME 2 (amplicon distribution 2024.2) as single-end sequences using a manifest file. Sequence quality was assessed with qiime demux summarize, and denoising was performed with the qiime dada2 plugin (v2024.2.0) to generate an amplicon sequence variant (ASV) table, representative sequences, and denoising statistics. Chimeric sequences were removed during the DADA2 workflow. Feature tables and representative sequences were summarized in QIIME 2 and exported for downstream analyses. Taxonomic assignment was performed in QIIME 2 using the feature-classifier classify-sklearn method with a Naive Bayes classifier trained on the Greengenes2 2022.10 backbone database. To match the amplified region, reference reads were first extracted using the 338F/806R primer pair, then restricted to bacterial sequences, and finally used to train a bacteria-specific classifier. Taxonomic bar plots were generated in QIIME 2 using the resulting taxonomy annotations. A phylogenetic tree was constructed from representative sequences using the align-to-tree-mafft-fasttree pipeline, and rooted trees were exported for downstream analyses.

### 2.3. Statistical Analysis

All statistical analyses were conducted in R (v4.5.0). Alpha diversity was evaluated using the Shannon diversity index and the Chao1 richness estimator. The effects of host species, season, and their interaction on alpha diversity were tested using general linear models, followed by pairwise comparisons where appropriate.

Because 16S rRNA amplicon sequencing data are compositional, beta diversity was analyzed within an Aitchison log-ratio framework rather than by applying conventional distance metrics directly to count tables. ASV counts were matched to metadata by SampleID and filtered to retain features present in at least 10% of samples. Zeros were replaced using Bayesian-multiplicative replacement implemented in the zCompositions package (v1.5.0-5), and the resulting table was transformed using the centered log-ratio (CLR) transformation in the compositions package (v2.0-9). Principal component analysis (PCA) was then performed on the CLR-transformed data using prcomp (v4.5.0), and the first two components were visualized with 95% confidence ellipses. Aitchison distances were calculated as Euclidean distances in CLR space. The effects of species, season, and species × season interaction on gut microbial community composition were tested using PERMANOVA (vegan package, v2.7-2) (adonis2, vegan, 999 permutations). Homogeneity of multivariate dispersion was additionally evaluated using PERMDISP.

Differential abundance analysis at the genus level was performed using ANCOM-BC2. Two comparison schemes were used: (i) seasonal contrasts within each host species (winter vs. summer), and (ii) interspecific contrasts within each season. No additional covariates were included in the ANCOM-BC2 models. *p* values were adjusted for multiple testing using the Benjamini–Hochberg false discovery rate (FDR), and taxa with adjusted q values below 0.05 were considered significant. Log-fold changes (LFCs) and 95% confidence intervals were visualized using ggplot2 (v4.0.1). Because summer and winter samples were collected from different individuals, all samples were treated as independent biological replicates, and no repeated-measures adjustment was applied.

Predicted functional profiles were inferred from the ASV table using PICRUSt2 (v2.5.3). Functional predictions were summarized at KEGG level 2 and compared between summer and winter within each host species. Ordination of predicted functional profiles was performed by PCA, and differences in the relative abundances of major KEGG level 2 categories between seasons were assessed within each species. Because these analyses were based on 16S rRNA gene data, all functional results were interpreted as inferred functional potential rather than direct measurements of functional activity. As the online PICRUSt2 workflow used in this study did not provide NSTI output, NSTI values were unavailable for evaluating reference genome representation, and the inferred functional profiles should therefore be interpreted with appropriate caution.

## 3. Results

### 3.1. Overview of the Sequencing Data

After DADA2 denoising and chimera removal, 41 samples were retained for downstream analyses. Across all samples, a total of 2,622,782 input reads were processed, of which 2,207,571 reads passed quality filtering. After denoising and removal of chimeric sequences, 951,957 non-chimeric reads were retained, with an average of 23,218 reads per sample. A total of 6491 ASVs were identified from the 16S rRNA gene sequencing data. Rarefaction curves approached saturation for all samples, indicating that the sequencing depth was sufficient to capture the majority of bacterial diversity (Figure S2).

### 3.2. Gut Microbiota Diversity Analysis

**Figure 1:**
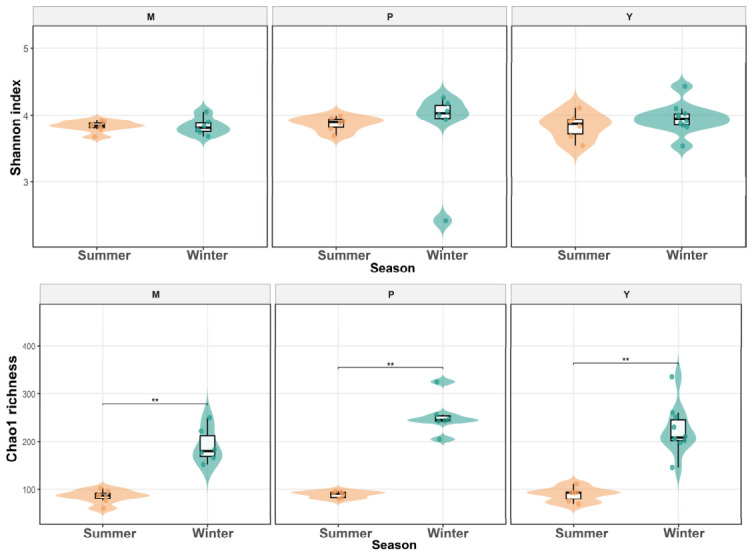
Seasonal variation in gut microbial alpha diversity across three host species. Gut microbial alpha diversity was assessed using the Shannon diversity index and the Chao1 richness estimator for mouflon (M), argali (P), and blue sheep (Y) in summer, and their corresponding winter groups (WM, WP, and WY). (* *p* < 0.05; ** *p* < 0.01; *** *p* < 0.001).

Alpha diversity analyses showed that seasonal effects were evident for richness-related diversity, whereas Shannon diversity did not vary significantly among species, between seasons, or through their interaction. For Shannon diversity, neither species (F_2,35_ = 0.18, *p* = 0.840, ηp^2^ = 0.010), season (F_1,35_ = 0.04, *p* = 0.845, ηp^2^ = 0.001), nor the species × season interaction (F_2,35_ = 0.32, *p* = 0.732, ηp^2^ = 0.018) had significant effects (Table 1). Pairwise comparisons likewise detected no significant seasonal differences within any host species and no significant species differences within either season (all *p* > 0.05, Figure 1). In contrast, Chao1 richness was strongly influenced by season (F_1,35_ = 159.17, *p* < 0.001, ηp^2^ = 0.820), whereas the effects of species (F_2,35_ = 2.99, *p* = 0.063, ηp^2^ = 0.146) and the species × season interaction (F_2,35_ = 2.31, *p* = 0.114, ηp^2^ = 0.117) were not statistically significant (Table 1). Pairwise comparisons showed that Chao1 richness was significantly lower in winter than in summer in mouflon, argali, and blue sheep (all *p* < 0.001, Figure 1). Species richness did not differ among host species in summer (all *p* > 0.05), whereas in winter mouflon showed lower richness than argali (*p* < 0.01), while the other pairwise comparisons were not significant. Overall, these results indicate that seasonal variation primarily affected richness rather than Shannon diversity, with a consistent winter decline in Chao1 across all three host species.

**Figure 2:**
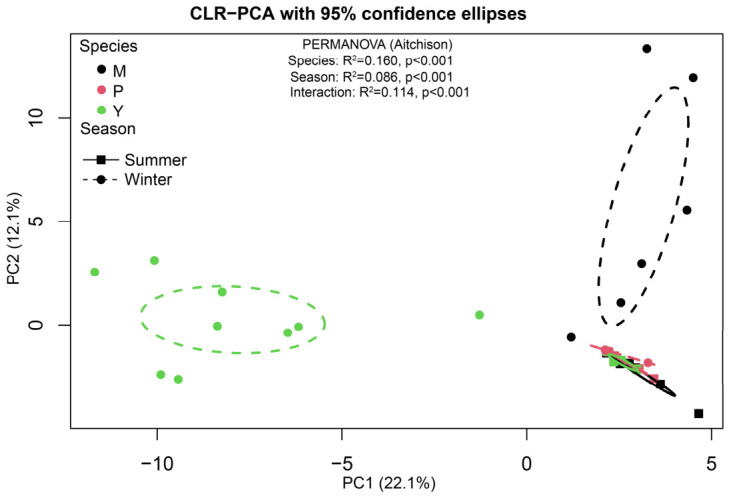
Compositional beta-diversity of gut microbiota across species and seasons based on CLR-transformed ASV data. M, P, and Y represent summer mouflon, argali, and blue sheep, respectively.

The compositional beta-diversity (CoDA) analysis revealed significant differences in gut microbiota composition across host species and seasons (Figure 2). The PCA based on the CLR-transformed data showed that the samples were relatively close across species in summer, whereas clearer separation was observed in winter. Seasonal shifts were evident in mouflon and blue sheep, but the directions of these shifts differed in the ordination space. In contrast, argali showed relatively greater overlap between summer and winter samples. The PERMANOVA on Aitchison distances confirmed significant effects of species, season, and their interaction (species × season) on gut microbial community composition (all *p* = 0.001), indicating that both host species and season shaped gut microbiota composition and that seasonal changes were species-dependent. However, PERMDISP detected significant heterogeneity in dispersion for season (F_1,32_ = 15.87, *p* < 0.001), species (F_2,31_ = 6.88, *p* < 0.01), and species × season groups (F_5,28_ = 5.01, *p* < 0.01). In particular, winter samples showed greater dispersion than summer samples. These results indicate that the observed beta-diversity differences should be interpreted with caution, as they may reflect both shifts in group centroids and differences in within-group variability.

### 3.3. Gut Microbiota Composition

All fecal samples from mouflon, argali, and blue sheep contained microbial communities spanning 11 phyla, 14 classes, 44 orders, 59 families, and 75 genera.

**Figure 3:**
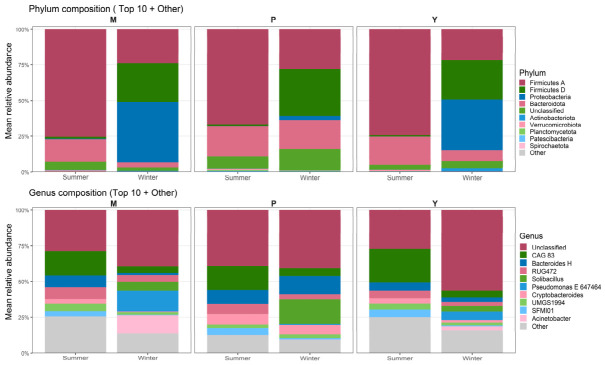
The relative abundance of gut microbial composition at the phylum and genus level. M, P, and Y represent summer mouflon, argali, and blue sheep, respectively.

At the phylum level (Figure 3; Figure S3), the gut microbiota of all three species was dominated in summer by Firmicutes_A, which accounted for 75.5% in mouflon, 66.6% in argali, and 74.4% in blue sheep, followed by Bacteroidota (M: 15.7%, P: 21.2%, Y: 19.5%). In winter, pronounced compositional shifts were evident in mouflon and blue sheep, characterized by marked increases in Proteobacteria (M: 0.4% → 42.2%; Y: 0.1% → 35.5%) together with declines in Firmicutes_A (M: 75.5% → 24.0%; Y: 74.4% → 21.6%) and Bacteroidota (M: 15.7% → 3.9%; Y: 19.5% → 7.7%). These winter shifts were also accompanied by a strong increase in Firmicutes_D (M: 1.2% → 27.0%; Y: 0.8% → 27.6%). In contrast, argali exhibited a comparatively more stable phylum-level profile across seasons, with Bacteroidota remaining similar between summer and winter (21.2% vs. 20.1%), a more moderate decline in Firmicutes_A (66.6% → 28.2%), and only a limited increase in Proteobacteria (0.1% → 3.0%), although Firmicutes_D also increased in winter (1.0% → 32.5%) (Figure 3; Figure S3). Consistently, the Firmicutes/Bacteroidota (F/B) ratio increased in winter groups, particularly in mouflon, whereas argali showed comparatively smaller seasonal changes (Figure S3).

At the genus level (Figure 3), all groups contained a high proportion of unclassified taxa, but the dominant classified genera varied markedly across species and seasons. Summer communities were characterized by relatively high abundances of CAG_83 (M: 17.0%; P: 16.7%; Y: 23.9%), Bacteroides_H (M: 8.1%; P: 10.1%; Y: 5.6%), RUG472 (M: 8.5%; P: 6.8%; Y: 5.1%), and UMGS1994 (M: 5.2%; P: 2.2%; Y: 4.2%), together with Cryptobacteroides in argali and blue sheep (P: 7.7%; Y: 4.0%). Winter communities diverged among host species. In mouflon, winter samples were characterized by strong increases in Pseudomonas_E_647464 (14.5%), Acinetobacter (12.6%), and Solibacillus (6.2%), taxa that were nearly absent in summer. In blue sheep, winter communities also showed clear increases in Pseudomonas_E_647464 (6.0%), Acinetobacter (2.5%), and Solibacillus (4.0%), together with a marked rise in the proportion of unclassified taxa (26.9% → 56.4%). In contrast, the winter shift in argali was less pronounced and was mainly characterized by increased Solibacillus (0.01% → 17.3%) and Bacteroides_H (10.1% → 12.6%), while most other dominant genera remained within a comparatively similar abundance range (Figure 3).

These seasonal patterns were further summarized by the heatmap of the top genera based on group means (Figure S4), which broadly separated winter groups from summer groups. This separation was driven mainly by a few genera showing the strongest seasonal contrasts, especially Acinetobacter, Pseudomonas_E_647464, Enterococcus_H_360604, Vulcanibacillus, and Solibacillus, all of which showed higher signals in winter than in summer.

**Figure 4:**
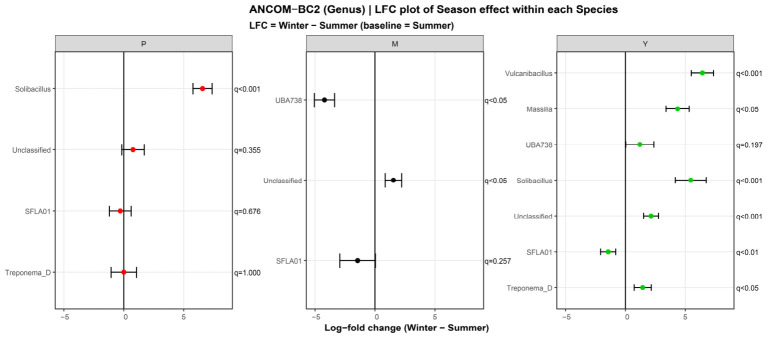
Within-species seasonal shifts in genus-level abundance inferred by ANCOM-BC2. M, P, and Y denote mouflon, argali, and blue sheep, respectively.

ANCOM-BC2 detected genus-level seasonal shifts within each host species (Figure 4). In argali (P), only Solibacillus was enriched in winter. In mouflon (M), winter samples showed a decrease in UBA738. Blue sheep (Y) showed a broader seasonal shift: Vulcanibacillus, Solibacillus, Massilia, and Treponema_D were enriched in winter, whereas SFLA01 was more abundant in summer.

We then compared host species within each season. To clarify pairwise contrasts, we re-ran ANCOM-BC2 with argali as the reference group (Figure S5). In summer, species differences were limited. Only Clostridioides_A was enriched in mouflon relative to argali, and no significant genus-level difference was detected between blue sheep and argali in this comparison. In winter, both mouflon and blue sheep differed more clearly from argali. Relative to argali, mouflon had higher abundances of Acinetobacter, Enterococcus_H_360604, Clostridium_F, and Paraprevotella, but lower abundances of Bacteroides_H and Cryptobacteroides. Blue sheep also differed from argali in winter, with higher abundances of Acinetobacter, Enterococcus_H_360604, Staphylococcus, and Pseudomonas_E_647464, and lower abundances of Solibacillus and Bacteroides_H.

We further summarized the three-species contrasts within each season using ANCOM-BC2 (Figure S6). Four genera differed significantly among species in summer, compared with 14 in winter. In summer, the main differences were driven by genera that were more abundant in mouflon, especially Clostridioides_A and RUG754, whereas Cryptobacteroides was relatively enriched in argali. In winter, a different set of genera separated the species. Acinetobacter remained enriched in mouflon relative to both argali and blue sheep, while Bacteroides_H and Cryptobacteroides were depleted in mouflon and comparatively higher in the other species. Additional genera, including UBA738, UBA3857, and Sodaliphilus, also contributed to winter differentiation.

### 3.4. Predicted Functional Profiles of the Gut Microbiota in Mouflon, Argali, and Blue Sheep

**Figure 5:**
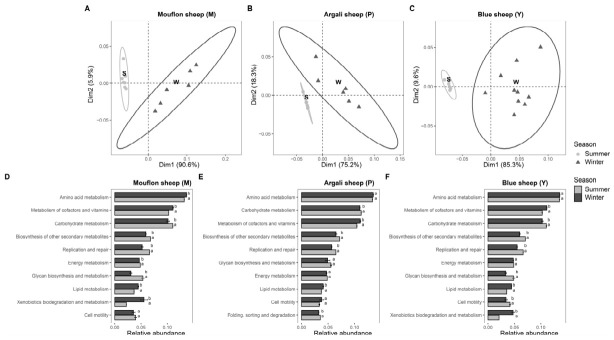
Predicted functional profiles of the gut microbiota inferred by PICRUSt2 within each host species across seasons. (**A**–**C**) PCA of KEGG level 2 predicted functional profiles in mouflon (**A**), argali (**B**), and blue sheep (**C**), showing the separation between summer and winter samples within each species. (**D**–**F**) Relative abundances of predicted KEGG level 2 functional categories inferred using PICRUSt2 in mouflon (**D**), argali (**E**), and blue sheep (**F**) across seasons. Different letters indicate significant differences between summer and winter within each species.

We used PICRUSt2 to infer the functional potential of the gut microbiota. The PCA revealed clear seasonal separation of predicted functional profiles within each host species (Figure 5A–C). In mouflon, argali, and blue sheep, summer samples formed relatively tight clusters, whereas winter samples were more dispersed and were separated from summer samples mainly along Dim1. This pattern indicates that seasonal shifts in predicted functional structure occurred in all three host species, although the extent of winter dispersion differed among them. Across all three species, the dominant predicted functional categories were broadly similar, with amino acid metabolism, metabolism of cofactors and vitamins, and carbohydrate metabolism showing the highest predicted relative abundances (Figure 5D–F). In mouflon, significant seasonal differences were detected in the predicted relative abundances of several functional categories. Summer samples showed relatively higher predicted abundances of pathways associated with amino acid metabolism, carbohydrate metabolism, biosynthesis of other secondary metabolites, replication and repair, and glycan biosynthesis and metabolism, whereas winter samples showed a higher relative abundance of predicted pathways related to the metabolism of cofactors and vitamins, lipid metabolism, and xenobiotics biodegradation and metabolism. In argali, seasonal shifts were more limited and mainly involved higher winter predicted abundances of pathways associated with metabolism of cofactors and vitamins, lipid metabolism, and folding, sorting and degradation, together with relatively higher summer predicted abundances of pathways associated with carbohydrate metabolism, biosynthesis of other secondary metabolites, and replication and repair. In blue sheep, winter samples showed higher predicted abundances of pathways associated with lipid metabolism, cell motility, and xenobiotics biodegradation and metabolism, whereas summer samples were characterized by higher predicted abundances of pathways associated with metabolism of cofactors and vitamins, carbohydrate metabolism, biosynthesis of other secondary metabolites, replication and repair, and glycan biosynthesis and metabolism. Overall, these results indicate that the predicted functional profiles of the gut microbiota shifted between summer and winter within each host species, but the specific functional categories contributing to seasonal differentiation differed among mouflon, argali, and blue sheep.

## 4. Discussion

A host’s gut microbiota is closely linked to its physiology, nutritional status, and environmental adaptation [23]. Even under captive conditions, where animals are maintained on the same diet, gut microbial communities can remain dynamic because factors other than diet, including ambient temperature, photoperiod, management regime, and host background, continue to shape microbial assembly [24,25,26]. In the present study, mouflon, argali, and blue sheep were maintained under the same feeding conditions, yet their gut microbiota still exhibited marked seasonal restructuring. Collectively, our results indicate that seasonal change remained the primary source of microbiome reorganization in captivity, but that this reorganization was not uniform across hosts. Summer communities were relatively close across the three species, whereas winter communities became more clearly differentiated, especially in mouflon and blue sheep. Thus, rather than indicating a single common winter microbiome state, the present results support a host-dependent seasonal response under captive conditions [26,27].

At the alpha-diversity level, the clearest seasonal pattern in our study was a winter decline in richness rather than a parallel loss of overall diversity. Chao1 richness decreased in winter across all three species, whereas Shannon diversity remained comparatively stable, indicating that seasonal change in this system affected richness more strongly than evenness. This pattern differs from that reported in captive forest and alpine musk deer, where gut microbial alpha diversity was higher in the cold season [14], suggesting that seasonal responses of the microbiome are not universal even among captive herbivores. Because all three sheep species in our study were maintained on the same diet, it is less likely that these winter changes can be attributed solely to seasonal shifts in food composition. Instead, seasonal host physiology and environmental cues such as temperature and photoperiod are more plausible drivers. This interpretation is consistent with experimental evidence that repeated temperature fluctuations can reshape gut microbiota and host metabolic plasticity, and with studies showing that seasonal changes in light-dark cycle and ambient temperature can alter the gut microbiome even under an unchanged dietary regimen [28,29,30]. Therefore, the winter decline in richness observed here may reflect seasonal physiological filtering rather than simple dietary turnover.

The beta-diversity results reinforce this view, but they also require caution. PCA based on CLR-transformed data showed that the three species were relatively close in summer, whereas winter samples became more clearly separated, particularly in mouflon and blue sheep. At the same time, PERMDISP indicated greater winter dispersion, suggesting that winter divergence reflects not only centroid shifts but also increased within-group variability. Even with this caveat, the overall pattern is clear: seasonal restructuring was stronger in winter, but it did not drive all hosts in the same direction. This finding is broadly consistent with other vertebrate studies showing that seasonal change can restructure both microbial composition and inferred function, while the magnitude and direction of response depend on host context [31,32]. In yaks and Tibetan sheep, for example, seasonal diet explained more variation than host species [27], whereas broader comparative analyses across vertebrates suggest that host evolutionary history and diet each contribute to different components of microbial diversity [26]. Our results fit between these two views: season strongly restructured the microbiota, but host identity still shaped how that restructuring unfolded.

At the phylum level, summer communities in all three species were broadly similar, being dominated by Firmicutes_A and Bacteroidota. Winter, however, brought much stronger taxonomic restructuring in mouflon and blue sheep. In both species, winter was associated with marked increases in Proteobacteria and Firmicutes_D, together with declines in Firmicutes_A and Bacteroidota. Argali followed a different seasonal trajectory: although Firmicutes_D also increased substantially, Proteobacteria remained comparatively low and Bacteroidota changed less. This distinction is important, because it indicates that argali did not remain unchanged in winter, but rather underwent a narrower and taxonomically different form of seasonal restructuring. The direction of change in the Firmicutes/Bacteroidota balance is broadly consistent with previous reports in captive musk deer and wild blue sheep, where seasonal shifts in dominant fermentative taxa were also interpreted as reflecting altered digestive demands under changing environmental conditions [14,15]. In herbivorous vertebrates, gut microbes contribute substantially to nutrient production and conservation, including fermentation of plant polysaccharides and generation of short-chain fatty acids [23,33]. However, the physiological implications of phylum-level shifts should not be overstated in the absence of direct metabolomic or physiological measurements.

The winter expansion of Proteobacteria in mouflon and blue sheep is one of the most striking findings of the present study and should be interpreted cautiously. In several systems, an increase in Proteobacteria has been associated with microbial instability or dysbiosis-related states, but it should not be treated as direct evidence of disease [34,35]. This caution is especially important here, because 16S rRNA amplicon data cannot distinguish harmless, environmentally associated, and potentially pathogenic members within the same higher-level taxonomic group. The genus-level results reinforce this point. ANCOM-BC2 and compositional summaries showed that winter restructuring involved repeated enrichment of several Bacillales/Planococcaceae-associated lineages and Proteobacteria genera, including Acinetobacter- and Pseudomonas-related taxa, especially in mouflon and blue sheep. Some Acinetobacter species are recognized as clinical opportunists in other contexts, but that evidence cannot be transferred directly to wildlife fecal microbiota [36]. In the present study, these taxa are therefore better interpreted as markers of winter-associated community restructuring than as evidence of pathogenic processes.

At the genus level, our results further indicate that winter divergence was host-dependent rather than uniform. Relative to summer, interspecific differences were limited in summer but became much more pronounced in winter, and within-species seasonal contrasts were narrower in argali than in blue sheep, with mouflon showing an intermediate pattern. This result fits the broader view that environmental filtering and host filtering operate simultaneously, and that seasonal challenge may expose host-specific microbial responses more strongly in some species than in others [26]. This interpretation is also ecologically plausible for argali and blue sheep. These two species are sympatric across large parts of the Qinghai-Tibet Plateau, yet they differ in habitat use, anti-predator strategy, and dietary niche [37,38]. Recent work further suggests that gut microbiota and diet jointly contribute to ecological niche differentiation between argali and blue sheep [39]. Although our animals were maintained in captivity, such species-specific ecological and evolutionary legacies may still influence how each host responds to seasonal conditions. Thus, the narrower winter shift in argali should not be interpreted as absence of response, but rather as a more constrained or more selective seasonal reorganization.

The predicted functional profiles inferred by PICRUSt2 also shifted between summer and winter within each host species, indicating that seasonal restructuring was not restricted to taxonomy. Across species, the dominant predicted KEGG level 2 categories were broadly similar, particularly amino acid metabolism, carbohydrate metabolism, and metabolism of cofactors and vitamins. However, the categories contributing to seasonal change differed among hosts, suggesting that seasonal restructuring of microbial functional potential was context dependent. Similar seasonal changes in inferred function have been reported in captive musk deer, Tibetan macaques, and hibernating bats, where shifts in predicted carbohydrate, lipid, and energy metabolism accompanied seasonal changes in microbial composition [14,31,32]. In our dataset, these differences should be interpreted strictly as changes in inferred functional potential rather than direct evidence of in vivo metabolic activity. Even so, the taxonomic and functional results together support the view that the winter microbiome was reorganized in ways that may alter host-microbe metabolic interactions.

Several limitations should be acknowledged. First, this study used a cross-sectional design, and summer and winter samples were collected from different individuals; therefore, seasonal contrasts reflect between-group differences rather than repeated trajectories within the same hosts. Second, all animals were maintained under managed conditions and fed the same controlled diet, which reduces some environmental noise but also means that captivity-related factors may have contributed to microbiota structure. Studies in managed echidnas and red deer have shown that captivity, enclosure use, and feeding regime can substantially alter gut microbiota composition [24,25]. Third, the blue sheep were rescued individuals with prior health-related histories, and although this background was unavoidable, unmeasured physiological state may have contributed to some of the observed variation. Finally, the functional analyses were based on amplicon-derived prediction and should therefore be treated as hypothesis-generating rather than definitive evidence of metabolic activity. Future studies integrating metagenomics, metabolomics, host physiological indices, and repeated sampling of the same individuals will be essential for clarifying the mechanisms underlying seasonal microbiome restructuring in captive herbivores.

## 5. Conclusions

Overall, our results show that seasonal change strongly restructures the gut microbiota of captive mouflon, argali, and blue sheep, but that winter restructuring is best understood as host-dependent divergence rather than a single shared seasonal response. Summer communities remained relatively similar across species, whereas winter intensified taxonomic and inferred functional differentiation, especially in mouflon and blue sheep. These findings highlight that even under standardized captive feeding conditions, seasonal environmental cues and host-specific background jointly shape the gut microbiome.

## Figures and Tables

**Table 1:** Summary of two-way ANOVA results for gut microbial alpha diversity.

Alpha Diversity Index	Factor	df	F	*p*	ηp^2^
Shannon	Species	2, 35	0.175	0.840	0.010
Shannon	Season	1, 35	0.039	0.845	0.001
Shannon	Species × Season	2, 35	0.315	0.732	0.018
Chao1	Species	2, 35	2.987	0.063	0.146
Chao1	Season	1, 35	159.173	<0.001	0.820
Chao1	Species × Season	2, 35	2.311	0.114	0.117

## Data Availability

The metadata and raw sequence reads have been deposited in the NCBI SRA database (PRJNA1371417).

## Supplementary Materials

The following supporting information can be downloaded at https://www.mdpi.com/article/10.3390/ani16101437/s1: Table S1: Sample animal informations; Table S2: General information on 16S rRNA sequence data; Figure S1: Venn diagrams showing shared and unique ASVs among species and between seasons; Figure S2: Rank-abundance curves (A) and rarefaction curves (B) of each sample; Figure S3: Abundance analysis of gut microbiota at the phylum level (top 5), and the three sample groups compared **by** the Kruskal–Wallis non-parametric test; Figure S4: Heatmap of the top genera across species and seasons.; Figure S5: ANCOM-BC2 genus-level differential abundance by season using argali (P) as the reference group; Figure S6: ANCOM-BC2 genus-level differential abundance by season using mouflon (M) as the reference group.
