# Supplementary material for: Seasonal and Species-Specific Variations in Gut Microbiota of Wild Ungulates in Captivity
Source: Animals (Basel). 2026 May 8;16(10):1437. doi: 10.3390/ani16101437 (PMC13203217; doi:10.3390/ani16101437)
Supplement: Supplementary file 1 [file animals-16-01437-s001.zip › animals-4219508-supplementary.pdf]

Table S1 Sample animal informations. M, P, Y represent mouflon, argali, and blue sheep in the summer, while WM, WP, WY represent mouflon, argali, and blue sheep in the winter.

Groups	Name	Sex	Senson	Weight	Environment	Food
M	QMFLYa1	Male	Summer	70 kg	Captivity	Corn and alfalfa
	QMFLYa2	Male	Summer	60 kg	Captivity	Corn and alfalfa
	QMFLYa3	Male	Summer	60 kg	Captivity	Corn and alfalfa
	QMFLYa4	Male	Summer	50 kg	Captivity	Corn and alfalfa
	QMFLYb1	Female	Summer	50 kg	Captivity	Corn and alfalfa
	QMFLYb2	Female	Summer	40 kg	Captivity	Corn and alfalfa
	QMFLYb3	Female	Summer	40 kg	Captivity	Corn and alfalfa
P	QPYa1	Male	Summer	150 kg	Captivity	Corn and alfalfa
	QPYa2	Male	Summer	140 kg	Captivity	Corn and alfalfa
	QPYa3	Male	Summer	120 kg	Captivity	Corn and alfalfa
	QPYb1	Female	Summer	80 kg	Captivity	Corn and alfalfa
	QPYb2	Female	Summer	100 kg	Captivity	Corn and alfalfa
	QPYb3	Female	Summer	85 kg	Captivity	Corn and alfalfa
Y	QYYa1	Male	Summer	70 kg	Captivity	Corn and alfalfa
	QYYa2	Male	Summer	60 kg	Captivity	Corn and alfalfa
	QYYa3	Male	Summer	42 kg	Captivity	Corn and alfalfa
	QYYb1	Female	Summer	50 kg	Captivity	Corn and alfalfa
	QYYb2	Female	Summer	50 kg	Captivity	Corn and alfalfa
	QYYb3	Female	Summer	62 kg	Captivity	Corn and alfalfa
WM	WMFLYa1	Male	Winter	70 kg	Captivity	Corn and alfalfa
	WMFLYa2	Male	Winter	70 kg	Captivity	Corn and alfalfa
	WMFLYa3	Male	Winter	42 kg	Captivity	Corn and alfalfa
	WMFLYb1	Female	Winter	70 kg	Captivity	Corn and alfalfa
	WMFLYb2	Female	Winter	60 kg	Captivity	Corn and alfalfa
	WMFLYb3	Female	Winter	60 kg	Captivity	Corn and alfalfa
WP	WPYa1	Male	Winter	48 kg	Captivity	Corn and alfalfa
	WPYa2	Male	Winter	70 kg	Captivity	Corn and alfalfa
	WPYa3	Male	Winter	63 kg	Captivity	Corn and alfalfa
	WPYb1	Female	Winter	50 kg	Captivity	Corn and alfalfa
	WPYb2	Female	Winter	60 kg	Captivity	Corn and alfalfa
	WPYb3	Female	Winter	40 kg	Captivity	Corn and alfalfa
WY	WYYa1	Male	Winter	50 kg	Captivity	Corn and alfalfa
	WYYa2	Male	Winter	80 kg	Captivity	Corn and alfalfa
	WYYa3	Male	Winter	70 kg	Captivity	Corn and alfalfa
	WYYa4	Male	Winter	72 kg	Captivity	Corn and alfalfa
	WYYa5	Male	Winter	60 kg	Captivity	Corn and alfalfa
	WYYb1	Female	Winter	40 kg	Captivity	Corn and alfalfa
	WYYb2	Female	Winter	45 kg	Captivity	Corn and alfalfa
	WYYb3	Female	Winter	50 kg	Captivity	Corn and alfalfa
	WYYb4	Female	Winter	50 kg	Captivity	Corn and alfalfa

WYYb5	Female	Winter	50 kg	Captivity	Corn and alfalfa

Table S2 General information of 16S rRNA sequence data.

Group	Sample	concentration (ng/μl)	Barcode	Raw Tags	Effective Tags
M	QMFLYa1	45.2	ATCGTT	77606	76777
	QMFLYa2	40.6	AATATC	81337	80003
	QMFLYa3	70.2	AAGCTC	73891	72572
	QMFLYa4	57.6	TTCCAT	80984	80064
	QMFLYb1	27	TCTAGG	83846	82341
	QMFLYb2	67.6	CTATAC	92841	92247
	QMFLYb3	84.2	GTCCCA	91716	91339
P	QPYa1	55.8	ATCGCA	70706	70231
	QPYa2	61	TTACGA	79008	78650
	QPYa3	61.2	TGTTAT	79646	78975
	QPYb1	74.8	GCCATC	71617	70943
	QPYb2	74.4	TGTGTT	76056	74579
	QPYb3	50.6	TAGGAC	82173	81252
Y	QYYa1	93	TGGACG	81846	81180
	QYYa2	90.2	CGATGT	109997	109226
	QYYa3	72.2	TCCTGT	86808	85721
	QYYb1	58.6	GAAGGC	72579	71703
	QYYb2	100	ATGTCA	69531	69199
	QYYb3	112	CGGTTA	85396	84649
WM	WMFLYa1	1.96	AAGGTA	48190	44825
	WMFLYa2	4.94	ATCACG	44013	41375
	WMFLYa3	0.66	CGGATG	50612	47305
	WMFLYb1	10.09	GTGAAA	50254	46966
	WMFLYb2	9.44	ATCTTG	46018	42661
	WMFLYb3	12.86	TATCTG	41564	38977
WP	WPYa1	6.01	AGAACA	50410	45969
	WPYa2	11.07	GGTGTG	41052	38238
	WPYa3	3.16	AGAGAC	49690	45774
	WPYb1	6.16	TATGCA	55323	51563
	WPYb2	5.16	GTAACA	50357	46419
	WPYb3	3.8	GCGAGG	52452	46647
WY	WYYa1	12.22	CGATGT	50606	47147
	WYYa2	12.04	TCCTGT	54329	50572
	WYYa3	6.42	GAAGGC	46152	43380
	WYYa4	8.17	ATGTCA	55077	51323
	WYYa5	6.52	CGGTTA	53732	50110
	WYYb1	3.04	TGTTAT	44786	41684
	WYYb2	9.19	GCCATC	44696	42220
	WYYb3	14.06	TGTGTT	40740	37442
	WYYb4	12.44	TAGGAC	56840	53367
	WYYb5	3.18	TGGACG	48305	45049

Total	2,622,728	2,530,664

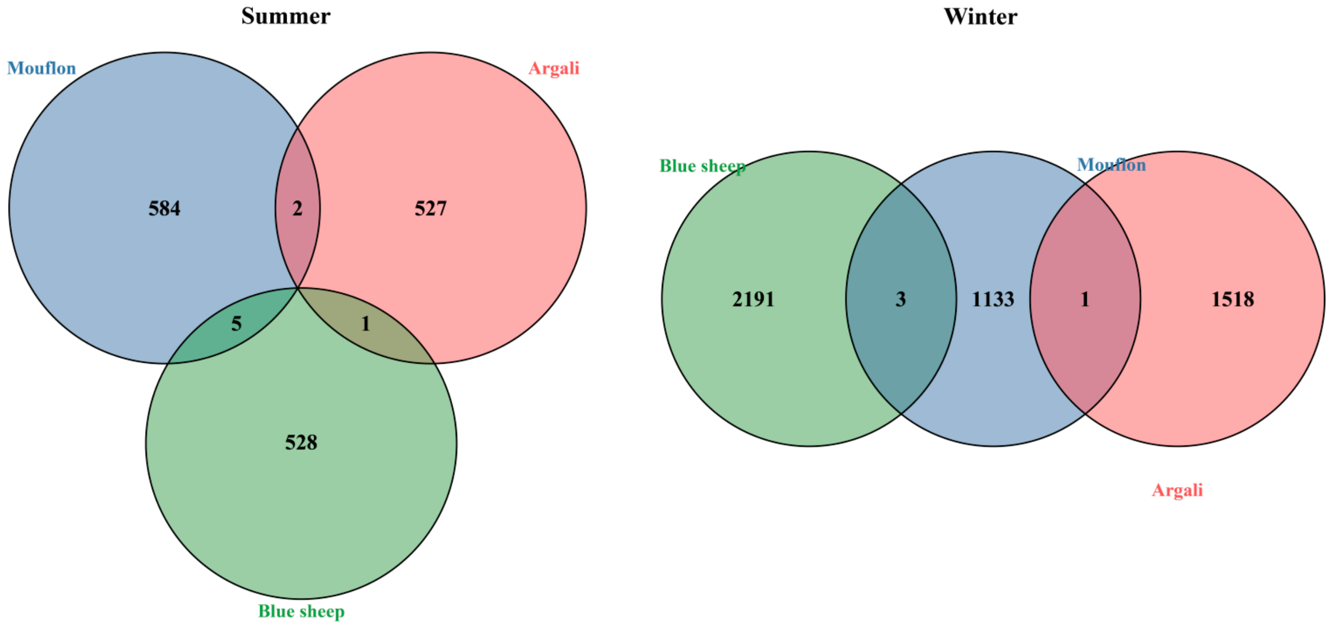

Figure S1 Venn diagrams showing shared and unique ASVs among species and between seasons.

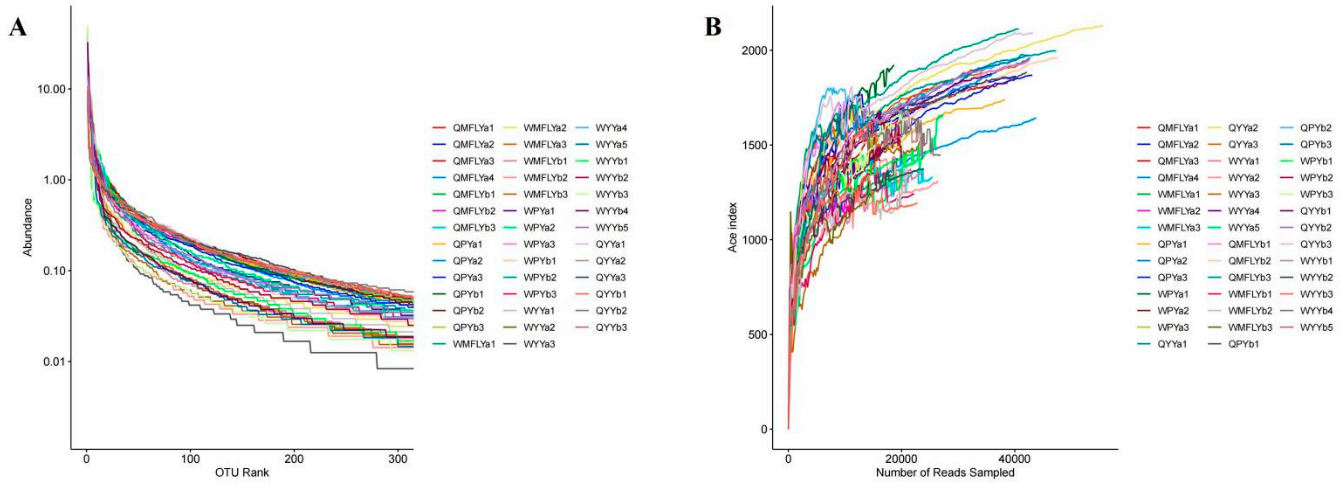

Figure S2 Rank-abundance curves (A) and rarefaction curves (B) of each sample. The rarefaction curves and species richness indices suggest that the sampling was thorough. In rank abundance curves, a broader range of curves indicates a higher relative species abundance, and a smoother curve on the y-axis signifies greater evenness among microbiota species in the fecal samples.

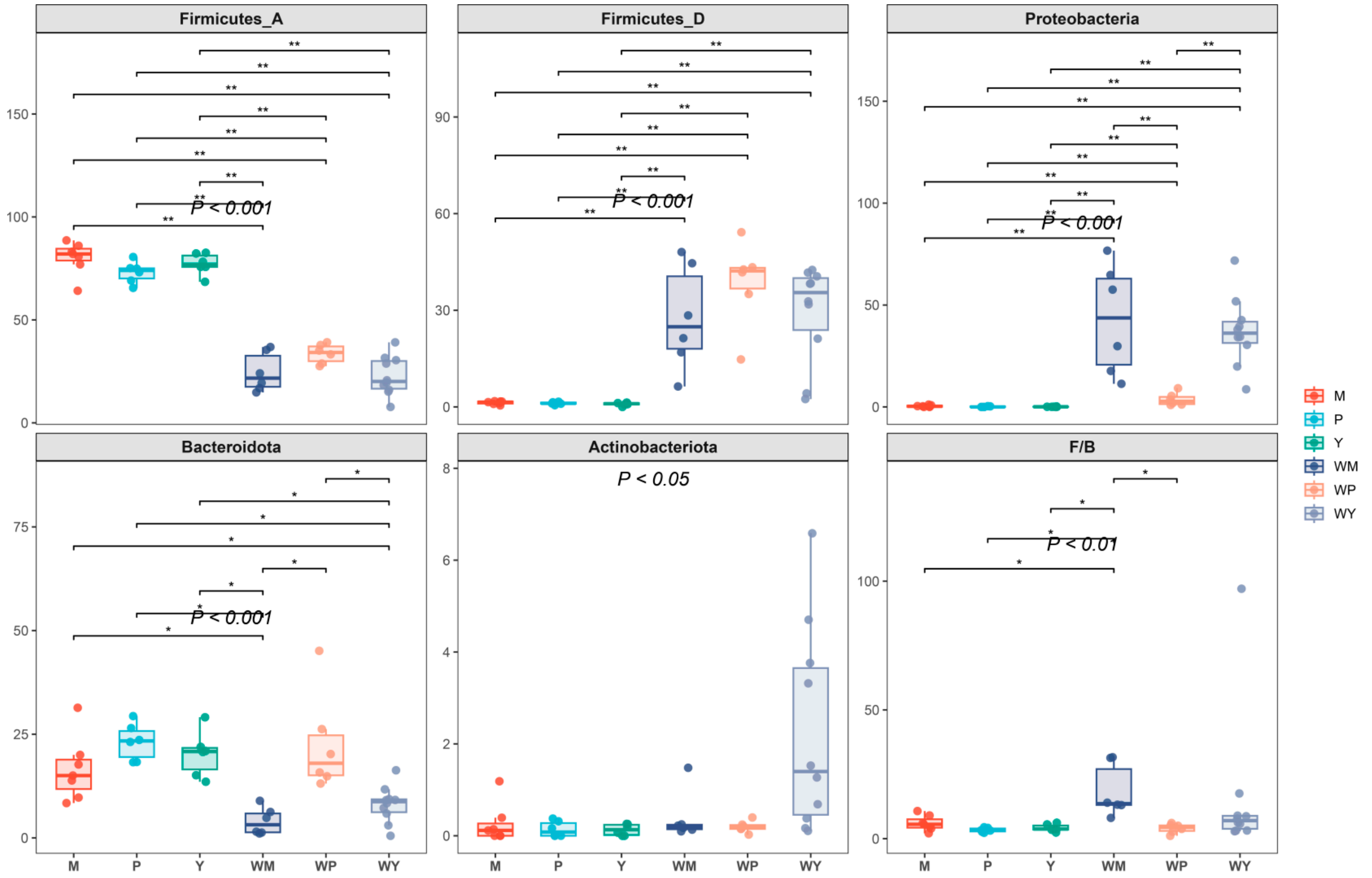

Figure S3 Abundance analysis of gut microbiota at the phylum level (top 5), and the three sample groups were compared by the Kruskal-Wallis nonparametric test. (**P* **< 0.05, **** *P* **< 0.01, and ***** *P* **< 0.001)**

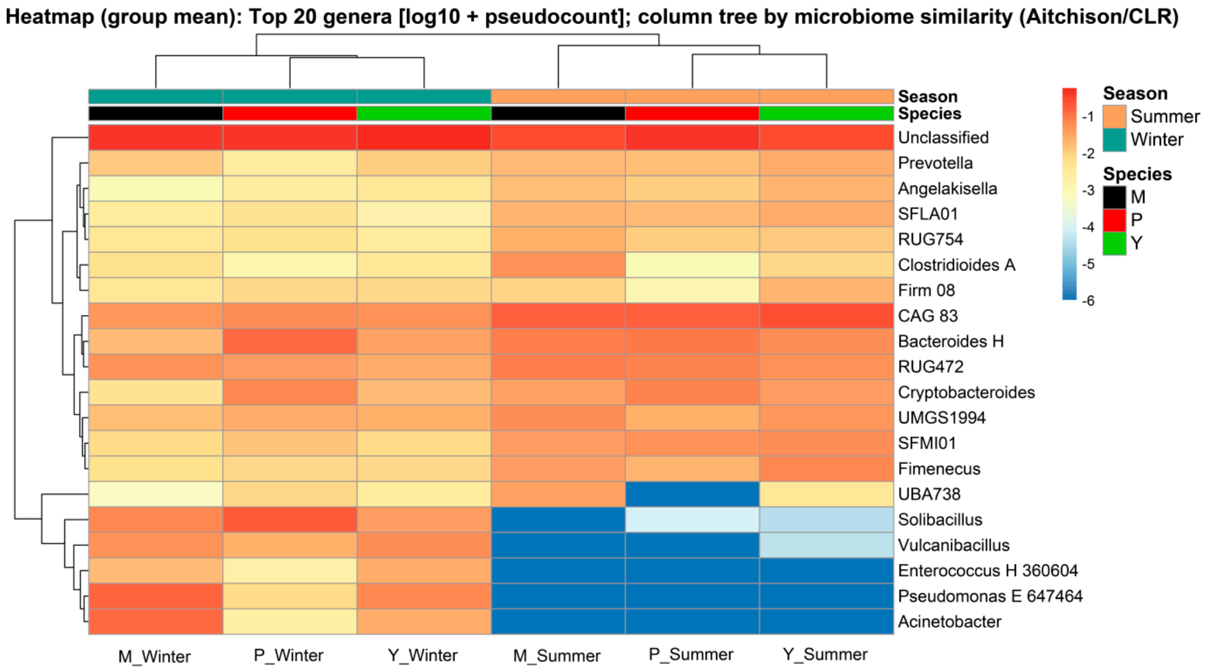

Figure S4 Heatmap of the top genera across species and seasons.

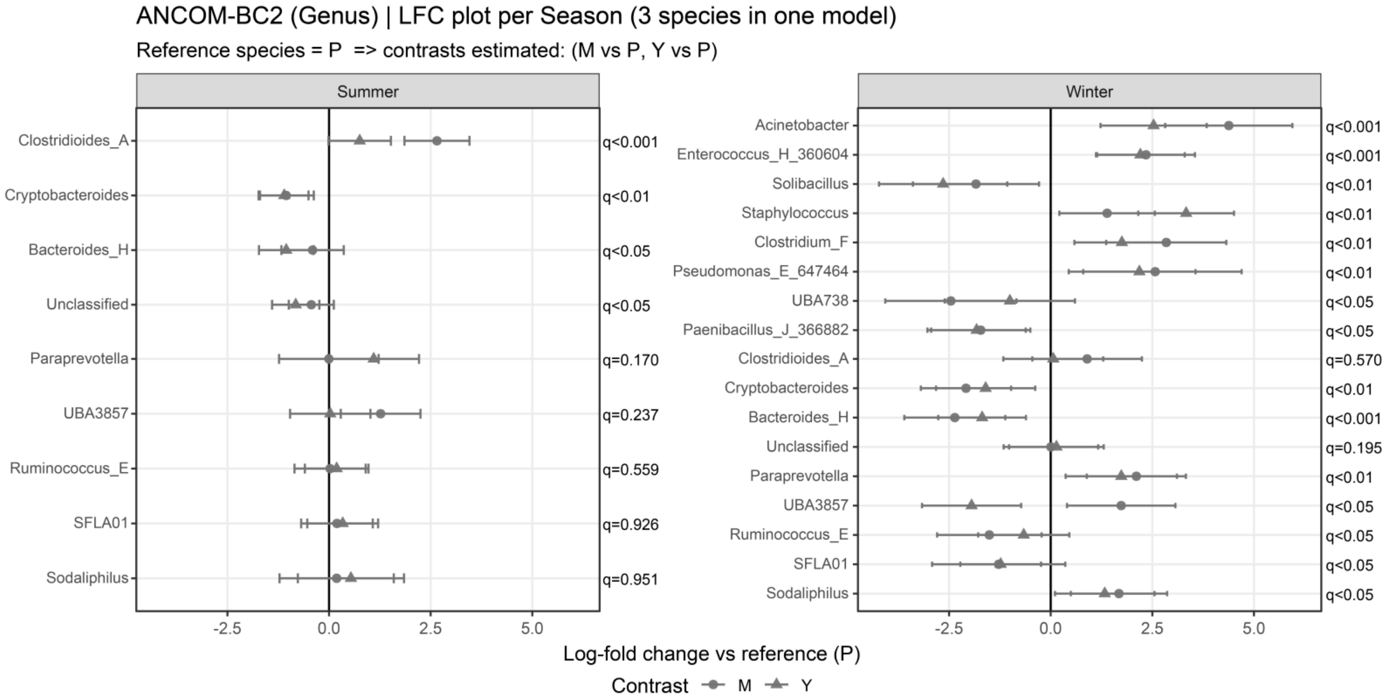

Figure S5 ANCOM-BC2 genus-level differential abundance by season using argali (P) as the reference group.

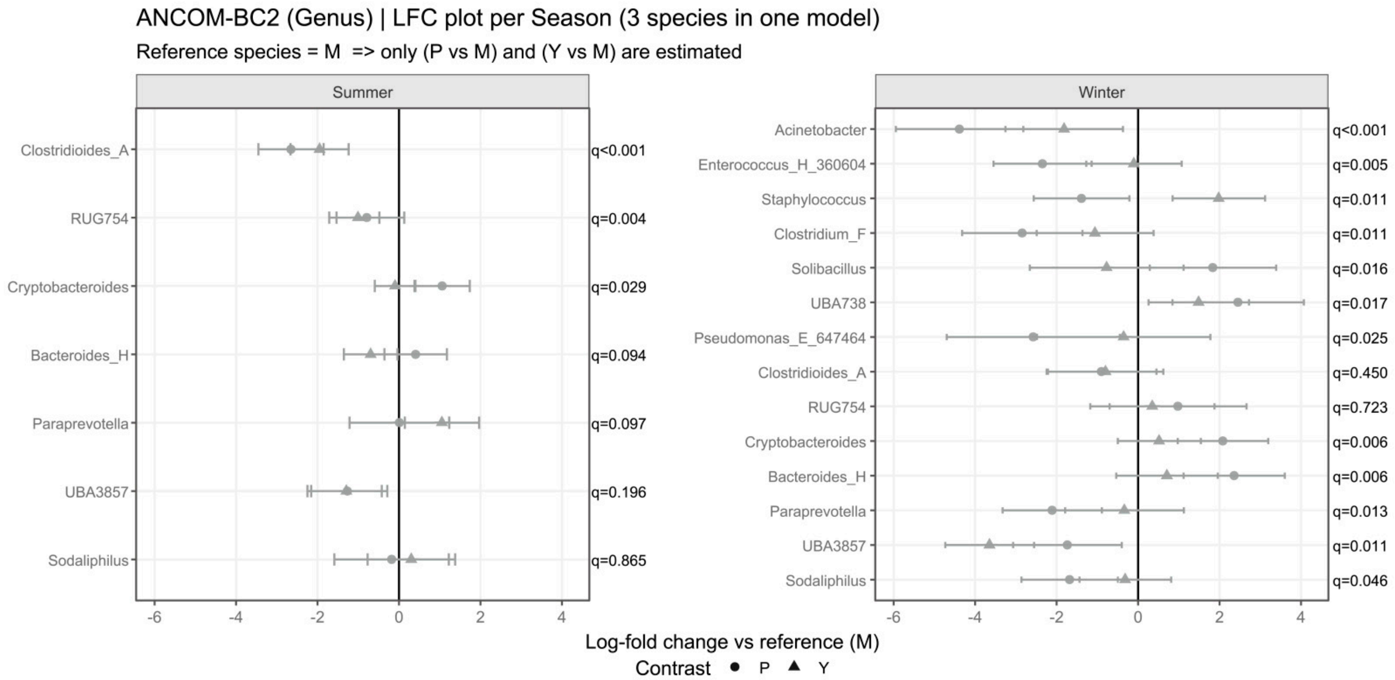

Figure S6 ANCOM-BC2 genus-level differential abundance by season using mouflon (M) as the reference group.
